# Reactions of citrinin with amino compounds modelling thermal food processing

**DOI:** 10.1007/s12550-024-00557-y

**Published:** 2024-09-19

**Authors:** Lea Brückner, Benedikt Cramer, Hans-Ulrich Humpf

**Affiliations:** Institute of Food Chemistry, University of Münster, Münster, Germany

**Keywords:** Citrinin, Mycotoxin, Food, Degradation, Stability, Biomonitoring

## Abstract

Citrinin (CIT) is a nephrotoxic mycotoxin, produced by several species of *Penicillium*, *Aspergillus*, and *Monascus*. The foodstuffs most frequently contaminated with CIT include cereals, cereal products, and red yeast rice. Studies on the occurrence of CIT in food have shown that the CIT concentrations in processed cereal-based products are generally lower than in unprocessed industry cereal samples. One possible explanation is the reaction of CIT with major food components such as carbohydrates or proteins to form modified CIT. Such modified forms of CIT are then hidden from conventional analyses, but it is possible that they are converted back into the parent mycotoxin during digestion. The aim of this study is therefore to investigate reactions of CIT with food matrix during thermal processes and to gain a deeper understanding of the degradation of CIT during food processing. In this study, we could demonstrate that CIT reacts with amino compounds such as proteins, under typical food processing conditions, leading to modified forms of CIT.

**Supplementary Information:**

The online version contains supplementary material available at 10.1007/s12550-024-00557-y.

## Introduction

Mycotoxins are secondary metabolites produced by fungi with toxic and sometimes carcinogenic effects. Structurally, mycotoxins are small molecules (< 1000 Da) with diverse chemical structures (Bennett and Klich 2003). Due to their toxic properties, they are undesirable in food and feed. Mycotoxins are mainly found in plant based food, but can also accumulate in animal products along the food chain (Bryden 2007).

**Fig. 1:**
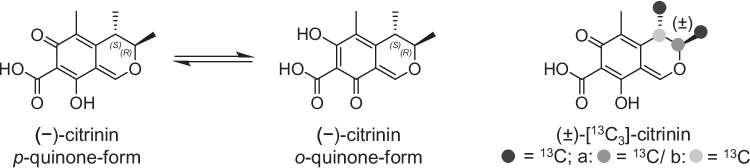
Chemical structure of CIT in its tautomeric forms *p*-quinone and *o*-quinone, as well as the chemical structure of the ^13^C_3_-labeled CIT

Citrinin (CIT, Fig. 1), which exists in two tautomeric forms, is a globally important contaminant of food. It is produced by several *Penicillium*, *Aspergillus*, and *Monascus* species. Thus, cereals and cereal-based products, fermented products such as red yeast rice, but also other products such as spices, vegetables, meat products, or cheese can be contaminated (Silva et al. 2020). CIT is nephrotoxic, teratogenic, and embryotoxic (Flajs and Peraica 2009; EFSA 2012). In terms of carcinogenicity, CIT is classified as “not classifiable as to its carcinogenicity to humans” (group 3) by the International Agency for Research on Cancer due to missing evidence of carcinogenicity (IARC 1986).

CIT is structurally related to and often accompanied by Ochratoxin A (OTA) (Vrabcheva et al. 2000). Moreover, CIT is discussed to increase the toxicity of OTA (Knecht et al. 2005). To that end, several studies have demonstrated additive or even synergistic toxic combinatorial effects of CIT and OTA (Speijers and Speijers 2004; Knecht et al. 2005; Flajs and Peraica 2009).

Compared to other mycotoxins, CIT has a moderate heat stability (Kabak 2009). CIT has been reported to completely decompose and detoxify under dry heating conditions at temperatures of 175 °C. At lower temperatures between 100 and 140 °C when water is added, reaction products like citrinin H1 and citrinin H2 can be formed (Kitabatake et al. 1991; Trivedi et al. 1993). Neely et al. (1972) report that the degradation of CIT in a solution of 95% ethanol or hexane begins at 60–70 °C. While citrinin H1 has an increased toxicity for HeLa cells, the toxicity of citrinin H2 for HeLa cells is lower than that of CIT (Hirota et al. 2002). Other known reaction products of CIT are the dimers dicitrinin A-D (Clark et al. 2006) as well as the decarboxylated form decarboxycitrinin (DCIT) (Clark et al. 2006; Devi et al. 2006).

However, studies investigating the degradation of CIT are limited and were solely performed with the pure compound (Hirota et al. 2002; Clark et al. 2006). The stability and degradation of CIT in food matrix are still largely uninvestigated.

A study funded by the European Food Safety Authority showed that the levels of CIT in processed cereal products are clearly much lower than those in non-processed samples (concentrations in the unprocessed samples ranged from 1.1 to 155 µg/kg, in the processed samples from 1.0 to 5.7 µg/kg). In addition, only 8% of all cereal products analyzed were contaminated with CIT (López Sánchez et al. 2017). The much lower CIT concentrations in processed samples demonstrate that food processing may have a strong impact on CIT levels, with the formation of modified forms of CIT during thermal food processing, investigated in this study, as a possible explanation.

The potential formation of modified forms of CIT is also supported by European human biomonitoring data. In German and Belgian cohorts, CIT and its main metabolite dihydrocitrinone (DH-CIT) were detectable in 59–82% and 6–84% of human urine samples indicating a high CIT exposure (Ali et al. 2015; Heyndrickx et al. 2015; Huybrechts et al. 2015). In the most recent study by Degen et al. (2022), either CIT or DH-CIT was detectable in all 179 and 142 urine samples from children and adults. This high human exposure does not correlate with the low contamination frequency (8%) in European food samples as described above. However, it has to be taken into account that the prevalence and concentrations of CIT in grains can fluctuate considerably from one year to another. Furthermore, recent exposure data from Switzerland show lower contamination levels for CIT and DH-CIT in a human cohort (2% and 0.1%), but as in this case, blood samples were analyzed; it is difficult to compare these percentages with those from urine analysis (Jaus et al. 2022).

It is known from various studies that the analyzed content of mycotoxins in a food matrix can be underestimated due to matrix associations of the respective mycotoxin (Rychlik et al. 2014). In the case of fumonisin B_1_, different functional groups (e.g., carboxyl or amino group) of the mycotoxin can react with matrix components such as hydroxy groups of polysaccharides or with hydroxy, amine, or thiol groups of proteins (Howard et al. 1998; Seefelder et al. 2001, 2003). Furthermore, it has been shown for OTA and T-2 toxin that these mycotoxins can covalently bind to matrix compounds such as polysaccharides during thermal food processing (Seefelder et al. 2003; Bittner et al. 2013; Kuchenbuch et al. 2019).

Since CIT has functional groups like the carboxyl or keto group, it can be assumed that CIT is able to react with food matrix components like polysaccharides or proteins as well. For this reason, we investigate in this study to what extent matrix-associated CIT is formed during thermal food processing, especially with regard to protein adducts.

## Materials and methods

### Chemicals and reagents

CIT (purity ≥ 98%) was purchased from Enzo Life Sciences (Lörrach, Germany). ^13^C_3_-labeled CIT (Fig. 1) was synthesized by Bergmann et al. (2018). DCIT (purity > 98%) was prepared as described below. *N*_α_-acetyl-L-lysine-methyl ester hydrochloride (98%), lisinopril ((S)-1-[N2-(1-carboxy-3-phenylpropyl)-lysyl-proline dihydrate) (certified reference material, pharmaceutical secondary standard), and gluten (from wheat, approx. 80% protein) were purchased from Sigma-Aldrich (Steinheim, Germany). Sodium chloride (≥ 99.5%) was purchased from Merck KGaA (Darmstadt, Germany). Potassium chloride (≥ 99.5%), and di-sodium hydrogen phosphate (≥ 99.5%) were purchased from Carl Roth (Karlsruhe, Germany). Potassium dihydrogen phosphate (≥ 99.5%) was purchased from Riedel-de Haën (Seelze, Germany). Pronase E (from *Streptomyces griseus*) was purchased from Roche Diagnostic (Mannheim, Germany). Purified water was generated using a Purelab Flex 2 system (Veolia Water Technologies, Celle, Germany). Acetonitrile (ACN) of LC–MS grade and Methanol (MeOH) of LC–MS grade were purchased from Fisher Scientific (Schwerte, Germany). Formic acid (> 99%) was purchased from Merck KGaA. Ammonia (25%, analytical reagent) was purchased from VWR Chemicals (Darmstadt, Germany). PBS buffer: 800 mg sodium chloride, 20 mg potassium chloride, 142 mg di-sodium hydrogen phosphate, and 24 mg potassium dihydrogen phosphate were dissolved in 100 mL purified water (pH 7.4).

### Isolation of DCIT from a large-scale heating experiment

Approximately 1 mg CIT was weighted into a 4-mL screw cap vial and dissolved in 2 mL purified water and then heated at 120 °C for 45 min. After freeze-drying of the reaction mixture, the residue was dissolved in 1 mL ACN/H_2_O (50/50, *v*/*v*). Purification was carried out using a semi-preparative HPLC–UV system consisting of a LC-NetII/ADC connector, two PU-2087 pumps, a UV-2075 detector (Jasco, Groß-Umstadt, Germany), a degasser (EGG 102.107, TechLab GmbH, Braunschweig, Germany), and a Rheodyne 7125 manual injector (optiLab Chromatographietechnik GmbH, Berlin, Germany). A Nucleodur C18 Gravity-SB column (250 × 10 mm, 5 μm, Macherey–Nagel, Düren, Germany) equipped with a universal C18 guard column (4.0 × 3 mm, Phenomenex, Aschaffenburg, Germany) was used for chromatographic separation. H_2_O + 0.1% formic acid was solvent A, and ACN + 0.1% formic acid was used as solvent B. The gradient was programmed as followed: 0 min 75% A, 1 min 75% A, 2 min 40% A, 15 min 0% A, 20 min 0% A, 20.1 min 75% A, and 25 min 75% A. The flow rate was set to 4 mL/min, and the column oven was operated at 40 °C. The detection wavelength was set to 295 nm, as most degradation products showed high intensities at this wavelength.

The purity of the isolated DCIT was determined using an HPLC–DAD-FLD-ELSD system consisting of a LC-NetII/ADC connector, two PU-2087 pumps, an autosampler (AS-2057 Plus), a DAD-detector (MD-2010), a fluorescence detector (FP-1520) (Jasco, Groß-Umstadt, Germany), and an evaporative light scattering detector (ELSD-LTS) (Shimadzu, Duisburg, Germany). The ELSD was set to a temperature of 50 °C and an air pressure of 350 kPa. A Nucleodur phenyl-hexyl-column (250 × 4 mm, 5 μm, Macherey–Nagel) equipped with a guard column with the same material (4.0 × 3 mm) was used for chromatographic separation. H_2_O + 1% formic acid was used as solvent A, and ACN + 0.1% formic acid was used as solvent B. The linear gradient was programmed as follows: 0 min 95% A, 1 min 95% A, 35 min 5% A, 37.5 min 5% A, 37.51 min 95% A, and 40 min 95% A. The flow rate was set to 1.4 mL/min.

The above described heating and isolation were carried out with a total amount of 5.57 mg CIT, leading to isolated 2.4 mg DCIT (yield: 52.3%). Purity of > 98% was determined by HPLC-ELSD. Fragmentation patterns (see Fig. S1, supplementary information) and UV–VIS maxima were in good accordance with literature (Clark et al. 2006; Devi et al. 2006). For a complete structural verification, NMR spectra (^1^H, ^13^C, gCOSY, gHSQC, gHMBC) in MeOH-*d4* were recorded on an Agilent DD2 600 MHz spectrometer (Agilent Technologies, Waldbronn, Germany) (see Fig. S3–Fig. S6 and Table S2, supplementary information).

Since both CIT and DCIT react with MeOH in a 1–4 Michael type nucleophilic addition, a methanol adduct of the *o*-quinone form of CIT is formed (Poupko et al. 1997) (compare Fig. S2, supplementary information). That is why in the ^1^H- and ^13^C-spectra, two signals for each hydrogen or carbon are visible. The reaction is reversible.

The molar extinction was determined in ACN by measurement at a wavelength of 327 nm and amounted to 5031 L mol^−1^cm^−1^.

### Spectroscopic data of Decarboxycitrinin (DCIT)

Yellow powder. *λ*_max_ (ACN): 205, 290.5, 327 nm. ^1^H NMR (600 MHz, MeOH-*d*_*4*_) δ_A_ 1.16 (d, *J* = 7.0 Hz, 3H, H-10), δ_B_ 1.19 (d, *J* = 6.7 Hz, 3H, H-10), δ_A_ 1.29 (d, *J* = 6.9 Hz, 3H, H-9), δ_B_ 1.31 (d, *J* = 6.4 Hz, 3H, H-9), δ_A_ 2.03 (s, 3H), δ_B_ 2.65 (dq, *J* = 7.4 Hz, 6.4 Hz, 1H, H-4), δ_A_ 2.72 (q, *J* = 7.0 Hz, 1H, H-4), δ_B_ 3.94 (dq, *J* = 7.4 Hz, 6.7 Hz, 1H, 3-H), δ_A_ 4.07 (q, *J* = 6.9 Hz, 1H, H-3), δ_A_ 5.42 (s, 1H, H-1), δ_B_ 5.52 (s, 1H, H-1), δ_B_ 6.21 (s, 1H, H-7), δ_A_ 6.24 (s, 1H, H-7), HRMS: *m*/*z* 207.1022 ([M + H]^+^ C_12_H_14_O_3_, calc. 207.1016, error 3.2 ppm) purity > 98%.

### Heating experiment—*N*_*α*_-acetyl-L-lysine-methyl ester hydrochloride

A stock solution of CIT (1 mg/mL in ACN) was prepared by dissolving 3 mg CIT in 3 mL of ACN. A stock solution of the model compound *N*_α_-acetyl-L-lysine-methyl ester hydrochloride (5 mg/mL) was prepared by dissolving 10 mg *N*_α_-acetyl-L-lysine-methyl ester hydrochloride in 2 mL ACN/water (1 + 1, *v*/*v*). In a 1.5-mL brown glass autosampler vial, 0.08 µmol CIT (20 µL stock solution) and 0.08 µmol *N*_α_-acetyl-L-lysine-methyl ester (40 µL stock solution) were pipetted and solvent removed at 40 °C under a stream of compressed air. The capped vial was heated at different temperatures (100 °C, 120 °C, 140 °C, 160 °C, 180 °C) and for different time periods (10, 30, 60 min). The obtained residue was dissolved in 500 µL of a mixture of water/ACN (80/20, *v*/*v*), vortexed for 30 s and then shaken for 30 min on a GFL type 3006 laboratory shaker (GFL, Gesellschaft für Labortechnik mbH, Burgwedel, Germany) (350 rpm) prior to analysis. All experiments were carried out in duplicate.

As control, 25 µL of the CIT stock solution (0.1 µmol) were pipetted in 1.5 mL brown glass vials without addition of the model compound. Further treatment and heating were carried out in the same way as with the addition of the model compound. Experiments with pure CIT were performed in triplicate.

To confirm the CIT-origin of identified reaction products, the heating experiment was repeated using ^13^C_3_-labeled CIT and the model compound *N*_α_-acetyl-L-lysine-methyl ester. A stock solution of ^13^C_3_-labeled CIT (1000 µg/mL in MeOH) was prepared by dissolving 1 mg ^13^C_3_-labeled CIT in 1 mL of MeOH. Ten µL of the ^13^C_3_-labeled CIT stock solution (0.04 µmol) and 20 µL of the *N*_α_-acetyl-L-lysine-methyl ester stock solution (0.4 µmol) were pipetted in 1.5-mL brown glass vials and dried at 40 °C under a stream of compressed air. The sample was heated at 160 °C for 10 min. The heated residue was dissolved as described above. All samples were analyzed using UHPLC-DAD-ESI-QTOF.

### Heating experiment: lisinopril

A stock solution of lisinopril (10 mg/mL) was prepared by dissolving 10 mg lisinopril in 1 mL of ACN/water (1 + 1, *v*/*v*). In a 1.5 mL brown glass vial, 0.04 µmol CIT (10 µL of the 1 mg/mL CIT stock solution in ACN) and 0.2 µmol lisinopril (10 µL of the stock solution) were pipetted and solvent removed at 40 °C under a stream of compressed air. The capped vial was heated at 140 °C for 10 min. The heated residue was dissolved in 500 µL of a mixture of water/ACN (80/20, *v*/*v*), vortexed for 30 s and shaken for 30 min on a GFL type 3006 laboratory shaker (GFL, Gesellschaft für Labortechnik mbH) (350 rpm). For a confirmation experiment, ^13^C_3_-labeled CIT was used in a heating experiment under the same conditions. All samples were analyzed using UHPLC-DAD-ESI-QTOF.

### Heating experiment: gluten

 Ten mg gluten was weighed in a 1.5-mL brown glass vial. Fifty µL of the 1 mg/mL CIT stock solution in ACN (0.2 µmol) and additionally 50 µL of purified water were added for easier mixing. The vial was gently shaken and solvents allowed to evaporate overnight. The next day, the dried residue was heated at 120 °C for 60 min. The vial was not sealed so that any remaining solvent and volatile heating products could evaporate. The heating experiment was done in the same way using ^13^C_3_-labeled CIT (control) instead of CIT. All experiments were carried out in triplicate.

After allowing the reaction product to cool down at room temperature, 500 µL of a pronase E solution (6.5 mg/mL in PBS-buffer) was added to the residue and the enzymatic digestion performed overnight at 37 °C while being gently shaken. The next day, the digested solution was centrifuged (14.000 × *g*, 5 min, rt), and the supernatant was pipetted onto a conditioned anion exchange solid phase extraction (SPE) cartridge (Oasis MAX 1 ml, 30 mg, Waters, Eschborn, Germany). To condition the SPE cartridge, 1 mL MeOH was first used, followed by 1 mL water. The column was rinsed with 1 mL water and eluted with 1 mL 70% MeOH, 1 mL 1% ammonia solution in MeOH, 500 µL MeOH, and finally with 1.5 mL 2% formic acid in MeOH. All fractions were collected and dried under a stream of nitrogen at 40 °C. The residues were each dissolved in 500 µL of a mixture of water/ACN (80/20, *v*/*v*) and analyzed using UHPLC-DAD-ESI-QTOF.

The amounts of CIT and DCIT were quantified by UHPLC-DAD-ESI-QTOF in a concentration range between 0.01 and 2 µg/mL (CIT *R*^2^ = 0.9989, DCIT *R*^2^ = 0.9989) by using UV detection at 335 nm. The concentration of the used CIT stock solution was calculated by using the molar extinction coefficient of 5490 L mol^−1^cm^−1^ (321 nm in MeOH) (El Adlouni et al. 2006). The concentration of the used DCIT stock solution was calculated by using the molar extinction coefficient of 5031 L mol^−1^cm^−1^ (327 nm in ACN) (see “Isolation of DCIT from a large-scale heating experiment”).

### Ultra-high-performance-liquid-chromatography coupled to DAD-detection and electrospray ionization quadrupole time-of-flight mass spectrometry (UHPLC-DAD-ESI-QTOF)

High resolution mass spectrometry analysis was carried out on an Impact II QTOF MS equipped with an Apollo II source (Bruker Daltonik, Bremen, Germany). The mass spectrometer was connected to an Elute HT Pump HPG 1300, an Elute Column Oven and a PAL HTC-xt auto sampler (CTC analytics, Zwingen, Switzerland).

The mass spectrometer was operated in positive ionization mode. The measurements were obtained in data dependent acquisition mode (Auto MS/MS) across a mass to charge range of *m*/*z* 30–1000 and a spectrum rate of either 6 Hz or 2 Hz. The following additional MS/MS parameters were used: end plate offset, 500 V; capillary, 4500 V; nebulizer, 2.0 bar; dry gas, 10 L/min; dry temperature, 220 °C; transfer time, 60 μs; and pre-pulse storage time, 10 μs.

A Nucleodur C18 Gravity-SB column (100 × 2 mm, 3 μm, Macherey–Nagel) equipped with a guard column with the same material (2.0 × 4 mm) was used for chromatographic separation. The temperature of the column oven was set to 40 °C. ACN + 1% formic acid was used as solvent A, and H_2_O + 1% formic acid was used as solvent B. The linear gradient was programmed as follows: 0 min 5% A, 1 min 5% A, 12 min 95% A, 15 min 95% A, 15.1 min 5% A, and 20 min 5% A. Flow rate was 0.35 mL/min and the injection volume 10 μL.

### Data processing and statistical analysis

Data acquisition and processing was performed with Compass Data Analysis 5.0 (Bruker). For statistical analysis the high-resolution mass spectrometry (HRMS) data was processed using MetaboScape 5.0 (Bruker).

For statistical analysis, a paired students *t*-test was performed. Fold change analysis combined with *p* values of the *t*-test analysis was used, to generate volcano plots using a significance level of 95%. Because zero *p* values are not applicable for *t*-test analysis, missing values were imputed for a user value. In this case, the lowest value found in the feature table (detection limit) was set as the default value. An intensity threshold was set to 10.000 cps so that features below that threshold are not displayed in the volcano plot.

## Results and discussion

### Model experiments with citrinin and* N*_α_-acetyl-L-lysine-methyl ester

In order to investigate the thermal stability of CIT in a simplified food matrix as well as to investigate possible reactions of CIT with amino functions as found in amino acids during heating, CIT was heated together with the model substance *N*_α_-acetyl-L-lysine-methyl ester at different temperatures and for different times. As a control group, pure CIT samples were heated under the same conditions. The samples were then analyzed using an UHPLC-DAD-ESI-QTOF instrument to identify and characterize newly formed reaction products and to determine the thermal degradation. The methoxylation of the carboxyl group and the acetylation of the α-amino group gives *N*_α_-acetyl-L-lysine-methyl ester chemical properties to mimic the reactivity of the amino acid lysine within a protein with only the ε-amino group being unprotected and therefore available for chemical reactions. The model substance has already been used in several other studies to investigate the influence of amino groups in proteins on the degradation of fumonisins, deoxynivalenol, nivalenol, T-2 toxin, and HT-2 toxin (Seefelder et al. 2003; Bretz et al. 2005, 2006; Beyer et al. 2009; Kuchenbuch et al. 2019).

**Fig. 2:**
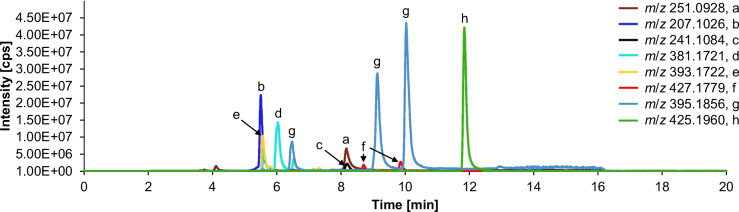
Combined extracted ion chromatogram of an UHPLC-QTOF analysis of CIT (**a**) heated for 10 min at 160 °C with water addition. Several degradation products were observed. Identified products: DCIT (**b**). Tentatively identified products: phenol A acid (**c**), dicitrinin A (**d**), dicitrinin C (**e**), and citrinin H1 (**f**). Structurally unknown degradation products with *m*/*z* 395.1856 ([M + H]^+^, C_24_H_26_O_5_) (**g**) and *m*/*z* 425.1960 ([M + H].^+^, C_25_H_28_O_6_) (**h**)

Analysis of the extracts from pure CIT heated at 120 °C and above showed the presence of the reported CIT degradation products, namely DCIT (*m*/*z* 207.1026), phenol A acid (*m*/*z* 241.1084), dicitrinin A (*m*/*z* 381.1721), dicitrinin C (*m*/*z* 393.1722), and citrinin H1 (*m*/*z *427.1779). Moreover, high amounts related to the peak height of two structurally unknown degradation products with *m*/*z* 395.1856 ([M + H]^+^, C_24_H_26_O_5_) and *m*/*z* 425.1960 ([M + H]^+^, C_25_H_28_O_6_) were observed (see Fig. 2).

**Fig. 3:**
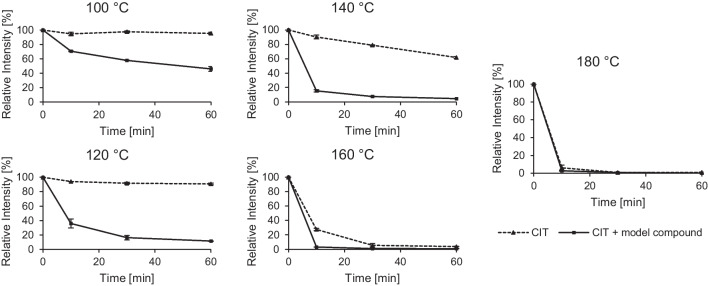
Thermal stability of CIT during heating with and without the matrix model compound (*N*_α_-acetyl-L-lysine-methyl ester) for different times (10, 30, 60 min) and temperatures (100, 120, 140, 160, 180 °C). The standard deviation, is given for analysis of pure CIT samples in triplicate, the mean deviation for analysis of CIT and model compound in duplicate, indicated by error bars

As shown in Fig. 3, pure CIT is stable at temperatures of 120 °C and below with no significant degradation. Above this temperature at 140 °C, a degradation of CIT occurs, with 90 ± 3% and 79 ± 0.6% of CIT remaining after 10 and 30 min respectively. At 160 °C, 27 ± 2% CIT are detectable after 10 min and a complete loss of CIT can be noted after 30 min.

Addition of the model compound *N*_α_-acetyl-L-lysine-methyl ester accelerates CIT degradation and leads already at 100 °C to only 70 ± 0.4% of the initial amount remaining after 10 min. After 30 min at 140 °C, CIT is almost completely degraded (only 8 ± 0.4% CIT left).

Temperatures above 160 °C led to the complete loss of CIT, even in the absence of the model compound, which is in good agreement with data from literature (Kitabatake et al. 1991; Trivedi et al. 1993).

Accelerated degradation in the presence of the lysine derivative is also described in the literature for other mycotoxins such as nivalenol, deoxynivalenol, T-2 toxin, and HT-2 toxin (Bretz et al. 2005, 2006; Kuchenbuch et al. 2019). The high degradation rate of CIT when heated with the lysine derivative may be due to alkaline catalysis by the ε-amino group as proposed by Bretz et al. (2006) for the degradation of deoxynivalenol or due to the formation of reaction products with this compound. The increased degradation rate of CIT in the presence of the model compound indicates that, also in the case of CIT, degradation products as well as reaction products with *N*_α_-acetyl-L-lysine-methyl ester could be formed. Indeed, chromatographic analysis of the sample extract revealed several signals for newly formed compounds, with the most abundant at a retention time of 6.7 min with *m*/*z* 419.2192, [M + H]^+^ (C_22_H_30_N_2_O_6,_ Fig. S7, supplementary information). The calculated molecular formula, which also contains two nitrogen atoms, indicates that CIT has reacted with the lysine derivative under the loss of a hydroxyl group. This is further supported by the recorded product ion spectrum of *m*/*z* 419.2169, where typical fragments of CIT (see Fig. S8), as well as fragments of the *N*_α_-acetyl-L-lysine-methyl ester can be assigned (see Fig. S7, supplementary information): The formed fragment with *m*/*z* 300.1592 and the sum formula C_18_H_22_NO_3_^+^ ([M + H]^+^) can be explained by the loss of the *N*-acetyl-α-amino-group as well as the carboxylic acid methyl ester group of the amino acid. Moreover, the loss of 200.1155 Da, leading to the fragment *m*/*z* 219.1014, equals to the neutral loss of the lysine derivative (C_9_H_16_N_2_O_3_). Other fragments such as the loss of water from the molecule, which also occurs during CIT fragmentation, and the loss of the carboxyl group through α-elimination (*m*/*z* 207.1014, [M + H]^+^, C_12_H_14_O_3_,) provide additional evidence that CIT is involved in the reaction (Demarque et al. 2016). Special importance for structure elucidation has the observed loss of CO (28 Da) to *m*/*z *391.2221. This elimination is characteristic from cyclic carbonyl compounds and was observed in quinones before (Bargen et al. 2012; Vessecchi et al. 2012; Demarque et al. 2016). Concerning the reaction of CIT with the ε-amino group of the lysine derivative, two reaction products might be favored, either the formation of an amide with the carboxylic acid group of CIT or the formation of an imine with the carbonyl function, both leading to the identical sum formula. In this case, the characteristic loss of the CO-group clearly indicates that the carboxyl group is involved, leading to the formation of an amide. If, on the other hand, the keto group would have reacted with the lysine derivative to form an imine, the elimination of the CO-group would not be possible.

To verify the identified compound as a reaction product of CIT and the lysine derivative, the experiment was repeated using ^13^C_3_-labeled CIT. The observed reaction product with a retention time of 6.7 min was exactly 3.0074 Da heavier (*m*/*z* 422.2266) than the previously observed reaction product of CIT and the lysine derivative (*m*/*z* 419.2192). This finding finally confirmed the reaction between CIT and the protein model substance *N*_α_-acetyl-L-lysine-methyl ester. Based on the observed reaction between CIT and the model compound, the next step was to investigate whether CIT is also capable of reacting with small peptides or whole proteins.

### Reaction of citrinin with a small peptide: lisinopril

**Fig. 4:**
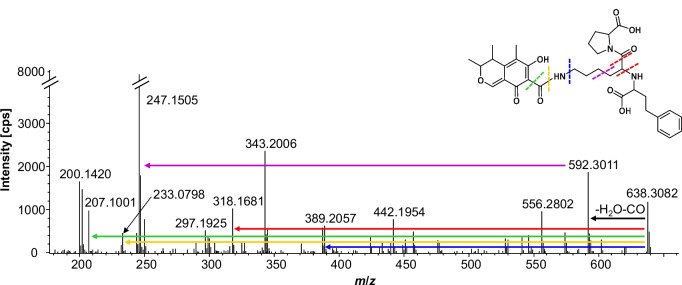
HRMS product ion spectrum of the reaction product of CIT and lisinopril with *m*/*z* 638.3082, retention time: 6.8 min, 44.1 eV ([M + H]^+^, C_34_H_43_N_3_O_9_). The postulated structure is given in the right corner of the fragment spectrum. Fragmentation pathways can be recognized by the arrows

To investigate the reaction between CIT and larger amino compounds than the previously used model compound during heating, an inexpensive commercially available small tripeptide “lisinopril” consisting of the natural α-amino acids (*S*)-proline, (*S*)-lysine, and an unnatural amino acid (*S*)-phenylalanine was heated together with CIT. As a control sample ^13^C_3_-labeled CIT was heated with lisinopril accordingly. All samples were analyzed using UHPLC-DAD-ESI-QTOF. The expected reaction products of CIT and lisinopril, which is formed under the loss of one water molecule, has a sum formula of C_34_H_43_N_3_O_9_ (see Fig. 4, [M + H]^+^, *m*/*z* 638.3072). Two single charged peaks with retention times of 6.8 and 8.7 min were observed in the extracted ion chromatogram of *m*/*z* 638.3082 ± 5 ppm, suggesting the formation of two isomers. Fragment spectra of both peaks were identical, and the mass error to the theoretically calculated mass of *m*/*z* 638.3072 was 1.5 ppm. In the control sample with ^13^C_3_-labeled CIT, the observed peaks with *m*/*z* 641.3155 were both exactly 3.0083 Da heavier than the reaction product *m*/*z* 638.3082 and retention time as well as fragment spectra matched with those of the unlabeled compounds. Therefore, it is confirmed that CIT can also react with small peptides.

In detail, the fragment spectrum of the lisinopril reaction product (see Fig. 4) with the parent ion *m*/*z* 638.3072 shows fragments typical for CIT (see Fig. S8, supplementary information). The subsequent neutral loss of the CO-group (− 46 Da) to *m*/*z* 592.3011 after the loss of water (− 18 Da) to *m*/*z *620.2941 should be mentioned. The fact that the keto group could also be split off here again indicates that the reaction product is rather the amide than the imine (see structure in Fig. 4). Further fragments that confirm that CIT was undoubtedly involved in the reaction are *m*/*z* 207.1001 (− 431 Da) and *m*/*z* 233.0798 (− 405 Da). The fragments have calculated sum formulas of C_13_H_15_O_3_^+^ and C_13_H_13_O_4_^+^ and correspond to the decarboxylated form of CIT (DCIT) as well as CIT minus water.

In addition, fragments such as *m*/*z* 318.1681 (− 320 Da), *m*/*z* 389.2057 (− 249 Da), and *m*/*z* 246.1475 (− 392 Da) can clearly be assigned to the fragmentation of lisinopril. In the case of *m*/*z* 389.2057, for example, (*S*)-proline and the unnatural homolog of (*S*)-phenylalanine are cleaved off.

### Reaction of citrinin with gluten

As CIT was found to react with a small peptide, the next step was to investigate whether reaction products can also be detected after heating CIT with a protein. Gluten was selected as a model compound as it is the most important storage protein in wheat but is also found in other cereals such as rye, barley, and oats. From a food technology perspective, gluten also plays a key role due to its viscoelastic properties (Biesiekierski 2017).

The analysis of potential reaction products of CIT with gluten required an extended sample preparation as the analysis of the intact protein by UHPLC-DAD-ESI-QTOF without enzymatic digestion is not possible. Thus, after heating the mixture of CIT and gluten, the protein was enzymatically digested using a mix of endopeptidases and exopeptidases from *Streptomyces griseus* (pronase E). The digested samples were purified by anion exchange SPE and the obtained fractions analyzed by UHPLC-DAD-ESI-QTOF.

**Fig. 5:**
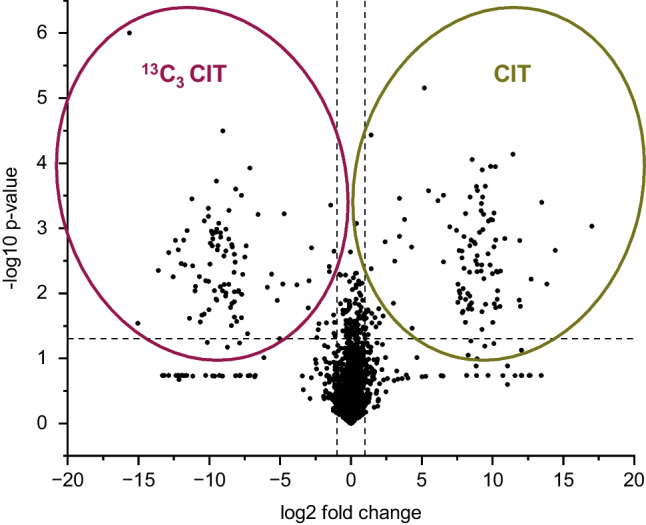
Volcano plot of the univariate data analysis of the heating experiment of CIT with gluten: on the right side features of the test group (CIT + gluten) and on the left side features of the control group (^13^C_3_-labeled CIT + gluten) are shown

For the identification of reaction products from CIT and amino acids, only the fraction eluting with acidified MeOH (1.5 mL 2% formic acid in MeOH) was studied in more detail, since potentially hydrophobic, carboxylic acid containing compounds are only eluted in this fraction from the polymeric bound ion exchange column. This also includes any kind of amino acid-bound reaction products of CIT. Instead of selective analysis for the reaction product of CIT with lysine, a more general approach for data analysis was chosen. Possible reaction products of CIT were identified by software-assisted comparison of the mass spectrometric data of the test group (CIT heated with gluten) with the control group (^13^C_3_-labeled CIT heated with gluten) based on the 3.0099 Da mass difference between the two CIT isotopomers. In a first step, the chromatograms obtained by UHPLC-DAD-ESI-QTOF were processed using MetaboScape 5.0 to generate a feature list of all detected peaks with their signal intensities, retention times, possible adduct ions, and fragment ions. The feature lists obtained from both experiments (with CIT or with ^13^C_3_-labeled CIT) carried out in triplicate were compared by univariate data analysis, such as fold change analysis and *t*-test analysis, which are suitable for detecting significant differences for a recognized feature between the two groups. The volcano plot combines the results of the fold change analysis and a *t*-test in one plot. In this way, differences between the two data sets were visualized. Features were considered potential reaction products of CIT and amino acid, as long as the following criteria were met: The feature had to have a fold change > 2, and the significance level was set at 95% (*p* value < 0.05). Peaks eluting with retention times after CIT (approximately 8 min) were excluded from processing as an increasing polarity of the reaction products due to the ionic character of the amino acids can be assumed. In addition, there had to be a feature in the test group (CIT heated with gluten) as well as a feature in the control group (^13^C_3_-labeled CIT heated with gluten) with the same retention time in a similar concentration that was 3.0099 Da heavier than in the test group. Features that were 6.0198 Da or 9.0297 Da heavier than the features in the test group were also examined in more detail as these could be reaction products containing two or three CIT molecules. Due to the powerful control group consisting of the isotope-labeled CIT heated with gluten, a further correction of the *p* values (e.g., the Benjamini–Hochberg procedure (Benjamini and Hochberg 1995)) was not performed, as the probability of false-positives was reduced to a minimum by the requirement for a corresponding feature with the exact mass difference in the control group. Additionally, only features with a calculated sum formula containing at least one nitrogen atom were accepted, while features known from a heating experiment in which CIT was heated without the addition of a model compound were excluded. Moreover, the product ion spectra (if available) were searched for specific fragments of CIT (*m*/*z* 233.0811, 215.0704, 205.0862, 191.0703, 147.0809). If one of the fragments was present, this further supports that the observed feature is a reaction product of CIT and may contain significant substructures of CIT. The latter may be of toxicological relevance as reaction products that contain the CIT backbone in an as intact state as possible may lead to comparable toxicity and increased probability of release of the intact CIT molecule during digestion. A total of 1217 features were identified for the test group in the volcano plot (see Fig. 5). After applying the described filter criteria, 15 features were identified as the most relevant potential reaction products of CIT with amino acids. The features shown in Table 1 are sorted according to their maximum signal intensity. Besides, the whole list of features and the results of the *t*-test can be found in the supplementary information (see Excel file). Unfortunately, due to the large number of potential reaction products that are formed and the small quantities available, it was not possible to isolate and further characterize these reaction products.

**Table 1:** Mass-to-charge ratios of the 15 features with the highest signal intensity, that are linked to CIT and possibly amino acids, formed in the heating experiment of CIT and gluten (samples were digested with pronase E after heating). Features are sorted according to their maximum intensity. The tentatively assigned sum formula, changes in comparison to the sum formula of CIT (C_13_H_14_O_5_), the calculated mass errors in ppm, as well as the mass-to-charge ratios of the control group (heating with ^13^C_3_ CIT) are given

*m*/*z*	Max. intensity	Assigned sum formula	Sum formula change	Δppm	-log10 (*p*-value)	log2 (fold change)		*m*/*z* (^13^C_3_ CIT)
324.1440	8.13E + 06	C_16_H_21_NO_6_	+ C_3_H_7_NO	0.40	2.66	14.43	327.1542^a^	
320.1491	4.90E + 06	C_17_H_21_NO_5_	+ C_4_H_7_N	0.33	2.14	7.56	323.1594^a^	
352.1389	3.93E + 06	C_17_H_21_NO_7_	+ C_4_H_7_NO_2_	0.58	3.40	13.47	355.1497^a^	
268.1543	2.63E + 06	C_14_H_21_NO_4_	+ CH_7_N -O	0.13	2.22	12.72	271.1644^a^	
622.2710	1.69E + 06	C_28_H_39_N_5_O_11_	+ C_15_H_25_N_5_O_6_	1.45	1.78	11.97	625.2818^a^	
525.2185	1.40E + 06	C_23_H_32_N_4_O_10_	+ C_10_H_18_N_4_O_5_	1.18	2.81	11.93	528.2221^a^	
326.1635	9.60E + 05	C_16_H_23_NO_6_	+ C_3_H_9_NO	4.48	3.13	3.79	329.1696^a^	
338.1961	6.69E + 05	C_18_H_27_NO_5_	+ C_5_H_13_N	0.16	2.84	10.87	341.2059^a^	
296.1494	5.63E + 05	C_15_H_21_NO_5_	+ C_2_H_7_N	0.53	3.58	5.49	299.1580^a^	
334.1645	4.96E + 05	C_18_H_23_NO_5_	+ C_5_H_9_N	1.13	2.04	10.36	337.1749^a^	
792.3670	4.78E + 05	C_37_H_53_N_5_O_14_	+ C_24_H_39_N_5_O_9_	1.03	2.34	10.29	795.3779^a^	
358.1495^b^	4.33E + 05	C_16_H_23_NO_8_	+ C_3_H_9_NO_3_	0.31	2.82	10.36	361.1595^a^	
653.2773	3.11E + 05	C_37_H_40_N_4_O_5_S	+ C_24_H_26_N_4_S	2.97	2.87	7.99	656.2881^a^	
481.1643	2.35E + 05	C_22_H_28_N_2_O_8_S	+ C_9_H_14_N_2_O_3_S	1.13	3.21	9.37	484.1745^a^	
738.3355	2.08E + 05	C_37_H_47_N_5_O_11_	+ C_24_H_33_N_5_O_6_	1.48	3.38	9.21	741.3466^a^	

^a^The control group has a mass difference of 3.0099 Da to the test group

^b^Due to instability of the compound, no product ion spectrum could be recorded

Binding with CIT may occur mainly via functional groups at the end of the amino acid side chain. For example, the basic amino group in the ε-position of lysine can react with acidic groups such as carboxyl groups of other substances. Other amino acids such as serine, threonine, or tyrosine carry hydroxyl groups, which are available for various reactions, e.g., esterification. Cysteine, on the other hand, contains a thiol group, which plays a decisive role in stabilizing the protein by forming disulfide bridges but may also act as a nucleophile in reactions.

**Fig. 6:**
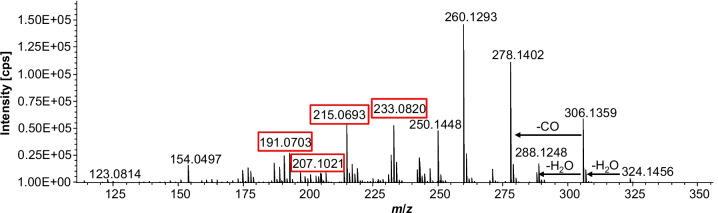
HRMS product ion spectrum of the feature with *m*/*z* 324.1456, retention time: 4.4 min, 30.6 eV ([M + H]^+^, C_16_H_21_NO_6_). The feature, that is presumably a thermal reaction product of CIT and an amino acid, is formed in the heating experiment of CIT with gluten (samples were digested with pronase E after heating). Typical CIT fragments are highlighted in red boxes, typical CIT fragmentation pathways like the loss of two water molecules and the loss of carbon monoxide can be recognized by the arrows

The most abundant signal intensity for a reaction product of CIT with gluten was recorded for *m*/*z* 324.1440 ([M + H]^+^, C_16_H_21_NO_6_). The corresponding product ion spectrum clearly confirmed that this is a reaction product from CIT (see Fig. 6). Besides the known neutral losses and fragments such as the double loss of water, the elimination of carbon monoxide also typical CIT fragments such as *m*/*z* 233.0820 (CIT-H_2_O), *m*/*z* 207.1021 (CIT-CO_2_), or *m*/*z* 191.0703 (CIT-C_2_H_4_O_2_) could be observed. The product ion spectra of the other reaction products identified shown in Table 1 behave similarly: Typical mass losses or characteristic CIT fragments can be identified in all product ion spectra (see Table S1, supplementary information).

The reaction product *m*/*z* 352.1389 with the sum formula C_17_H_21_NO_7_ would fit to the possible reaction product of CIT and threonine. Other reaction products including the reaction product of CIT with lysine that can be directly attributed to a reaction between CIT and a specific amino acid could not be identified. The reason for this might be that, in addition to the degradation of CIT, the thermal treatment also led to intramolecular modifications of the protein. A further explanation for the missing CIT-lysine reaction product could be the low lysine content in gluten, favoring other reactions (Rombouts et al. 2009).

In order to estimate the amount of modified CIT, the content of CIT and DCIT was quantified in the reaction mixture. No other degradation products besides DCIT were detected. Of the 50 µg CIT used, 10.0 ± 0.6% unreacted CIT could still be found, and 2.3 ± 0.2% were converted to DCIT. This indicates that up to 87.7% of the CIT could have reacted to so far unknown reaction products or are bound to gluten.

In principle, various mycotoxins and their metabolites or degradation products are able to react with proteins or form stable complexes (Hagelberg et al. 1989; Fliege and Metzler 1999; Schwerdt et al. 1999; Sueck et al. 2018; Csenki et al. 2023). Lysine and arginine are both positively charged amino acids and often react with other substances in proteins in particular, as their side chains are active and rise out of the protein complex (Sokalingam et al. 2012). A popular example is the reaction of aflatoxin B_1_, which forms covalent adducts with the ε-amino group of lysine in human serum albumin (Sabbioni et al. 1987; Gerdemann et al. 2022). CIT has also been described to form stable complexes with human serum albumin. H-bonds, salt bridges, and hydrophobic interactions are the main suspected binding forces (Poór et al. 2015).

There are also numerous examples of mycotoxins being bound to macromolecules in food matrix during thermal processes, although most of these studies relate to covalent bonds between sugars or starch and the respective mycotoxin (Bretz et al. 2005, 2006; Bittner et al. 2013; Kuchenbuch et al. 2019). For fumonisin B_1_, covalent bonds could also be demonstrated to proteins in model experiments using the model substance *N*_α_-acetyl-L-lysine-methyl ester (Seefelder et al. 2003).

Until now, there are no studies published showing that CIT forms covalent bonds with amino compounds or proteins in general during thermal processes. However, underestimations of CIT during food sample analysis have been reported due to analytical issues. Hou et al. (2021) have observed that the analysis of CIT in *Hongqu* (red fermented rice) leads to underestimation if only small amounts of acid are added to the extraction solvent. Various methods for sample preparation and the subsequent analysis of CIT in food are described, whereby the optimum conditions are always strongly dependent on the food matrix (Atapattu and Poole 2020). However, as official analytical methods show, a substantial quantity of acid such as 1% hydrochloric acid is used to achieve acceptable recovery rates when analyzing CIT (CEN EN 17203:^2021^; López Sánchez et al. 2017).

A clear distinction must be made between the underestimation of CIT due to an unsuitable sample preparation and extraction method (Atapattu and Poole 2020) and a more general underestimation of CIT exposure due to modified forms of CIT as shown in this study with the thermal reaction of CIT with amino acids (Rychlik et al. 2014). In particular, the latter may explain on the one hand why CIT levels are lower in processed food samples and on the other hand why CIT is found often in human urine samples, but is rarely conspicuous in food screening.

In this study, we could demonstrate that CIT is generally able to play a role in food as a modified mycotoxin. The heating experiment of CIT and gluten clearly showed that CIT reacts with parts of the protein leading to new bound forms of CIT that maintain important chemical substructures of CIT as all three ^13^C labels remained in the detected reaction product and the characteristic mass spectrometric fragments of CIT could also be observed in the product ion spectra. In addition to proteins, starch might also be a possible reaction partner for CIT.

From a toxicological point of view, the question remains to which extend the modified forms of CIT are released by the digestion of food and especially by the intestinal microbiota. This should be part of future studies and can be done, for example, by using the pig cecum model system, which has already been successfully used in our research group for modified mycotoxins like T-2 and HT-2 toxin to study their metabolism (Kasimir et al. 2020).

To support and supplement these studies on the reactions of CIT with amino compounds, the reactions of CIT with other macromolecules such as polysaccharides are currently under investigation. This and the reactions of CIT during food processing will be reported in future publications.

## Conclusion

Model experiments were performed to study the reaction of CIT with amino compounds during thermal processing of food samples. For this purpose, a lysine derivative, a small peptide, and a whole protein were heated with CIT, and the resulting reaction products were analyzed using untargeted HRMS. Reaction products from CIT and amino compounds were detected in all experiments. In particular, the feature list generated from the experiment with gluten can be used in the future to analyze cereal samples digested with pronase E for CIT adducts. In all cases, the reaction was confirmed with ^13^C_3_-labeled CIT.

These data suggest that modified forms of CIT such as thermal reaction products with amino acids or proteins might explain why generally lower CIT concentrations are found in processed food compared to raw materials and why CIT is frequently detected in human biomonitoring samples. However, the later aspect requires that modified forms of CIT are cleaved during digestion to liberate CIT. This has to be studied in further experiments.

## Supplementary Information

Below is the link to the electronic supplementary material.

Supplementary file1 (DOCX 259 KB)

Supplementary file2 (XLSX 311 KB)

## Data Availability

The authors declare that the data supporting the findings of this study are available within the paper and its  Supplementary Information files. Should any raw data files be needed in another format they are available from the  corresponding author upon reasonable request.

## Abbreviations

ACN: Acetonitrile
CIT: Citrinin
DCIT: Decarboxycitrinin
DH-CIT: Dihydrocitrinone
HRMS: High resolution mass spectrometry
MeOH: Methanol
OTA: Ochratoxin A
SPE: Solid phase extraction
UHPLC-DAD-ESI-QTOF: Ultra-high-performance-liquid-chromatography coupled to DAD-detection and electrospray ionization quadrupole time-of-flight mass spectrometry
