# Supplementary material for: Reactions of citrinin with amino compounds modelling thermal food processing
Source: Mycotoxin Res. 2024 Sep 19;40(4):709–20. doi: 10.1007/s12550-024-00557-y (PMC11480111; doi:10.1007/s12550-024-00557-y)
Supplement: Supplementary file 1 — Supplementary file1 (DOCX 259 KB) [file 12550_2024_557_MOESM1_ESM.docx]

**Supplementary material**

**Table S1:** HRMS product ion spectrum data of the features, that are linked to CIT and possibly amino acids, formed in the heating experiment of CIT and gluten (shown in Table 1). The exact mass of the parent ion, the applied collision energy and the exact masses of the corresponding major fragment ions are indicated and the relative signal intensity is given in brackets as a percentage. Major fragment ions are shown with a relative signal intensity higher than 10 %, with the exception of *m*/*z* 268.1543and *m*/*z* 326.1635, as fragment ions with a relative intensity higher than 3 % are shown here due to poor fragmentation. For the feature *m*/*z* 358.1495 no product ion spectrum could be recorded due to the instability of this compound over time.

| *m*/*z* of the parent ion | Major fragment ions  (Relative intensity is given in brackets as a percentage) |
| --- | --- |
| 320.1491  (EPI^+^, 30.5 eV) | 306.1347 (10), 303.1233 (13), 302.1757 (10), 285.1126 (13), 278.1388 (21), 274.1436 (62), 260.1285 (24), 259.1332 (10), 258.1204 (17), 257.1174 (100), 250.1438 (11), 247.1324 (16), 243.1017 (26), 233.1169 (12), 232.1065 (10), 231.1018 (48), 229.1224 (21), 219.1014 (10), 215.1068 (10), 201.0910 (17), 191.0708 (13) |
| 352.1389  (EPI^+^, 31.5 eV) | 306.1342 (73), 289.1074 (10), 278.1391 (16), 261.1313 (17), 260.1282 (100), 243.1016 (18), 233.1173 (33), 232.1334 (12), 217.0863 (13), 215.1067 (40), 205.0733 (10), 191.0702 (16), 187.1119 (14) |
| 268.1543  (EPI^+^, 19.2 eV) | 250.1437 (3), 223.0970 (7), 207.1019 (33), 205.0862 (100), 187.0759 (3), 177.0912 (10), 159.0806 (3) |
| 622.2710  (EPI^+^, 28.7 eV) | 372.1889 (100), 354.1781 (82), 257.1250 (53), 251.0919 (38), 244.1296 (33), 240.0985 (19), 116.0720 (11) |
| 525.2185  (EPI^+^, 25.8 eV) | 275.1358 (94), 258.1091 (100), 257.1251 (95), 251.0920 (36), 241.0825 (19), 240.0985 (10) |
| 326.1635  (EPI^+^, 20.7 eV) | 309.1528 (14), 308.1496 (80), 306.1336 (12), 291.1220 (5), 280.1659 (4), 278.1385 (7), 263.1468 (3), 262.1439 (15), 245.1166 (5), 219.1016 (3), 207.1021 (3) |
| 338.1961  (EPI^+^, 31.0 eV) | 206.0898 (16), 205.0862 (100), 187.0756 (19), 177.0914 (36), 159.0806 (23), 149.0961 (10) |
| 296.1494  (EPI^+^, 39.9 eV) | 280.1661 (12), 205.0859 (40), 190.0625 (60), 187.0752 (42), 177.0911 (68), 163.0750 (20), 162.0677 (18), 161.0598 (13), 160.0840 (11), 159.0804 (100), 149.0963 (44), 144.0573 (10), 135.0807 (14), 133.0648 (12), 131.0856 (78), 123.0819 (15), 121.1020 (23) |
| 334.1645  (EPI^+^, 40.9 eV) | 271.1326 (12), 261.1487 (22), 257.1167 (10), 246.1217 (15), 245.1172 (78), 233.1172 (26), 227.1065 (24), 219.1016 (100), 215.1065 (13), 201.0908 (15), 191.1067 (21), 189.0911 (15) |
| 792.3670  (EPI^+^, 53.8 eV) | 324.1443 (100), 306.1343 (99), 278.1390 (55), 245.1138 (20), 233.1173 (10) |
| 653.2773  (EPI^+^, 29.6 eV) | 403.1950 (100), 387.1715 (11), 386.1686 (60), 385.1846 (80), 369.1415 (26), 257.1252 (27), 251.0920 (19), 240.0982 (15), 147.0767 (34) |
| 481.1643  (EPI^+^, 24.5 eV) | 464.1398 (100), 302.1520 (13), 287.0927 (30), 279.2327 (10), 267.1205 (30), 257.1260 (13), 251.0914 (35), 244.1289 (22), 219.1032 (12) |
| 738.3355  (EPI^+^, 32.1 eV) | 488.2521 (100), 470.2417 (52), 373.1896 (15), 263.1396 (70), 251.0918 (33) |

**Table S2:** ^1^H NMR and ^13^C NMR chemical shifts of citrinin (δ_A_) and the citrinin-methanol adduct (δ_B_) in MeOH-*d4* respectively. For assignment of positions see Fig S2.

| Position | ^1^H NMR (600 MHz, MeOH-*d4*)  δ (ppm) | | | ^13^C NMR (151 MHz, MeOH-*d4*)  δ (ppm) | |
| --- | --- | --- | --- | --- | --- |
|  | | **δ_A_** | **δ_B_** | **δ_A_** | **δ_B_** |
| 1 | | 5.42 (s, 1H) | 5.52 (s, 1H) | 96.44 | 96.78 |
| 3 | | 4.07 (q, *J* = 6.9 Hz, 1H) | 3.94 (dq, *J* = 7.4 Hz, 6.7 Hz, 1H) | 74.87 | 71.34 |
| 4 | | 2.72 (q, *J* = 7.0 Hz, 1H) | 2.65 (dq, *J* = 7.4 Hz, 6.4 Hz, 1H) | 36.32 | 38.05 |
| 4a | | - | - | 139.10, 139.07 | 140.84, 140.81 |
| 5 | | - | - | 114.07 | 114.33 |
| 6 | | - | - | 157.60, 157.56 | 157.80, 157.77 |
| 7 | | 6.24 (s, 1H) | 6.21 (s, 1H) | 101.81 | 100.91 |
| 8 | | - | - | 154.61, 154.57 | 154.20, 154.15 |
| 8a | | - | - | 111.49 | 113.68 |
| 9 | | 1.29 (d, *J* = 6.9 Hz, 3H) | 1.31 (d, *J* = 6.4 Hz, 3H) | 21.84 | 20.80 |
| 10 | | 1.16 (d, *J* = 7.0 Hz, 3H) | 1.19 (d, *J* = 6.7 Hz, 3H) | 20.76 | 20.00 |
| 11 | | 2.03 (s, 3H) | 2.03 (s, 3H) | 11.94 | 10.08 |

**Fig. S1:** HRMS product ion spectrum of the isolated DCIT (*m*/*z* 207.1022), retention time: 5.4 min, 32.7 eV ([M+H]^+^, C_12_H_14_O_3_).

**Fig. S2:** Chemical structure of DCIT in its tautomeric forms *p*-quinone and *o*-quinone, as well as the formed methanol adduct in MeOH-*d4*.

**Fig. S3:** ^1^H-NMR of DCIT in MeOH-*d4*.

**Fig. S4:** ^1^H-NMR of DCIT in MeOH-*d4* (0-2.3 ppm).

**Fig. S5:** ^1^H-NMR of DCIT in MeOH-*d4* (2.2-6.4 ppm).

**Fig. S6:** ^13^C-NMR of DCIT in MeOH-*d4*.

**Fig. S7:** Top: Base peak chromatogram of the heating experiment of CIT and *N*_α_-acetyl-L-lysine-methyl ester. Below: HRMS product ion spectrum of the reaction product with *m*/*z* 419.2169, retention time: 6.8 min, 38.0 eV ([M+H]^+^, C_22_H_30_N_2_O_6_).

**Fig. S8:** HRMS product ion spectrum of CIT (*m*/*z* 251.0921), retention time: 8.2 min, 33.8 eV ([M+H]^+^, C_13_H_14_O_5_).
